# Understanding the Role of Layered Minerals in the Emergence and Preservation of Proto-Proteins and Detection of Traces of Early Life

**DOI:** 10.1021/acs.accounts.4c00173

**Published:** 2024-08-14

**Authors:** Sarah V. Stewart, Valentina Erastova

**Affiliations:** † School of Chemistry, University of Edinburgh, Joseph Black Building, David Brewster Road, Edinburgh EH9 3FJ, United Kingdom; ‡ UK Centre for Astrobiology, School of Physics and Astronomy, University of Edinburgh, James Clerk Maxwell Building, Peter Guthrie Tait Road, Edinburgh EH9 3FD, United Kingdom

## Abstract

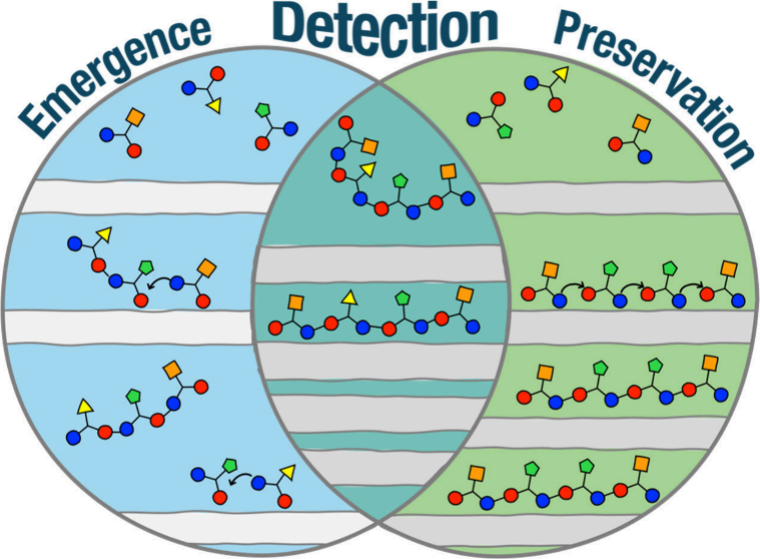

The origin of life remains one of the most profound mysteries in science. Over millennia, theories have evolved, yet the question persists: *How did life emerge from inanimate matter?* At its core, the study of life’s origin offers insights into our place in the universe and the nature of life itself. By delving into the chemical and geological processes that led to life’s emergence, scientists gain a deeper understanding of the fundamental principles that govern living systems. This knowledge not only expands our scientific understanding but also has profound implications for fields ranging from astrobiology to synthetic biology.

This research employs a multidisciplinary approach, combining a diverse array of techniques, from space missions to wet laboratory experiments to theoretical modeling. Investigations into the formation of the first proto-biomolecules are tailored to explore both the complex molecular processes that underpin life and the geological contexts in which these processes may have occurred. While laboratory experiments are aimed at mimicking the processes of early planets, not every process and sample is attainable. To this end, we demonstrate the use of molecular modeling techniques to complement experimental efforts and extraterrestrial missions. The simulations enable researchers to test hypotheses and explore scenarios that are difficult or impossible to replicate in the laboratory, bridging gaps in our understanding of prebiotic processes across vast time and space scales.

Minerals, particularly layered structures like clays and hydrotalcites, play diverse and pivotal roles in the origin of life. They concentrate organic species, catalyze polymerization reactions (such as peptide formation), and provide protective environments for the molecules. Minerals have also been suggested to have acted as primitive genetic materials. Nevertheless, they may lack the ability for long-term information replication. Instead, we suggest that minerals may act as transcribers of information encoded in environmental cyclic phenomena, such as tidal or seasonal changes. We argue that extensive protection of the produced polymer will immobilize it, making it inactive for any further function. Therefore, in order to generate a functional polymer, it is essential that it remains mobile and chemically active. Furthermore, we suggest a route to the identification of pseudobiosignatures, a polymer that was polymerized on the same mineral surface and consequently retained through overprotection.

This Account presents a comprehensive evaluation of the current understanding of the role of layered mineral surfaces on life’s origin and biosignature preservation. It highlights the complexity of mineral-organic interactions and proposes pathways for proto-biomolecule emergence and methods for identifying and interpreting potential biosignatures. Ultimately, the quest to uncover the origin of life continues to drive scientific exploration and innovation, offering profound insights into the fundamental nature of existence and our place in the universe.

## Key References

- Erastova, V.; Degiacomi, M. T.; Fraser, D. G.; Greenwell, H. C. Mineral Surface Chemistry Control for Origin of Prebiotic Peptides. *Nat. Commun.***2017**, *8* (1), 1–9.^1^*This modeling study investigates interactions of layered hydroxides with amino acids under early Earth conditions. It identifies a mechanism for prebiotic peptide formation, highlighting the importance of environmentally driven processes and open systems*.
- Grégoire, B.; Erastova, V.; Geatches, D. L.; Clark, S. J.; Greenwell, H. C.; Fraser, D. G. Insights into the Behavior of Biomolecules on the Early Earth: The Concentration of Aspartate by Layered Double Hydroxide Minerals. *Geochim. Cosmochim. Acta***2016**, *176*, 239–258.^2^*Combining laboratory experiments with classical and quantum molecular modeling techniques, this study characterizes interactions between amino acids and layered double hydroxides, highlighting routes for peptide formation*.
- Nuruzade, O.; Abdullayev, E.; Erastova, V. Organic-Mineral Interactions under Natural Conditions: A Computational Study of Flavone Adsorption on Smectite Clay. *J. Phys. Chem. C***2023**, *127* (27), 13167–13177.^3^*The molecular dynamics study of the adsorption and retention of small natural organic molecules in clay employs a realistic model of natural clay and ensures environmentally relevant conditions*.
- Zhao, R.; Xue, H.; Lu, S.; Greenwell, H. C.; Erastova, V. Revealing Crucial Effects of Reservoir Environment and Hydrocarbon Fractions on Fluid Behaviour in Kaolinite Pores. *Chem. Eng. J.***2024**, *489*, 151362.^4^*This modeling study examines the adsorption behavior of a broad selection of naturally occurring organic compounds on kaolinite’s two distinct basal surfaces and quantifies the effect of environmental conditions*.

For millennia, philosophers and scientists have attempted to determine the origin of life (OoL) on Earth. For ancient Greek philosophers, life originated by spontaneous generation from inert matter. It was not until 1668 that this idea was first challenged when Italian physician Francesco Redi showed that maggots came from the eggs of flies. However, spontaneous generation was not disregarded until 1859, when Louis Pasteur disproved it once and for all,^5^ and a biogenic theory of life was proposed. This rather recent shift in the theory of life’s origins is not surprising when we consider that biological evolution was only proposed in the middle of the 19th century, chemical evolution was not clearly discussed until the 1920s, and the basic molecular constituents of life (namely the structure of DNA) were only discovered in the 1950s.^5^ Research in this field has been a constant back and forth, where new things are discovered and old theories revisited. Despite the great advances in science over the years, the origin of life remains an enduring mystery.

In order to search for life’s origin, one must first define what life is. While this may at first seem a simple exercise, it is not trivial to find a definition that cannot also be applied to abiotic systems. However, a general, minimal, working definition of a living thing as an *open chemical system able to transfer its molecular information* (through self-reproduction) *and increase in complexity* (through evolution) is most commonly used today.^6^ Another definition, proposed by Gánti, focuses on the observable features of life: compartmentalization, metabolism, and information storage/transfer.^7^ Therefore, for life to emerge, all these features have to arise. Life-as-we-know-it is polymer-based: proteins, ribonucleic acids, carbohydrates, and lipids synergistically drive life’s processes. The question shared between many OoL theories is what abiotic processes would have created proto-metabolism and inheritance from simple abiotic starting materials.^8^

The famous Miller-Urey experiment demonstrated abiotic synthesis of amino acids, the simplest building blocks of life, under early Earth conditions (as believed at the time).^9^ Later, amino acids were also identified on meteorites and in space, confirming their omnipresence.^10^ The most prevalent OoL theories all postulate that water is essential to life and that life began in liquid water; ranging from shallow pools (Darwin’s “warm little pond” theory), to hot, briny, organic-dense oceans (Oparin-Haldane “primordial soup” hypothesis), to deep oceans (some hydrothermal vent theories).^11^ However, in dilute solutions, even if the reactants come into contact, the formation of larger molecules is often inhibited due to hydrolysis out-competing condensation polymerization reactions, and so a method by which to concentrate or aggregate these molecules is also needed.^12^ Many of the main theories of the OoL, thus, include the use of geological minerals, such as clays, to concentrate, confine, and catalyze the reactions.^12,13^

While many OoL theories exist, the difficulty arises in the assumptions made and their experimental validation. Due to the Earth’s plate tectonics, geochemical records of the prebiotic Earth are scarce, while laboratory testing relies on the ever-changing knowledge of early conditions. However, some clues for ancient life have been preserved in the geological record from the Archean Eon (4.0–2.5 billion years ago (BYA)).^14^ There are several independent lines of evidence that tell us life was present on Earth 3.5 BYA, but any older fossils claiming to show evidence of life have been contested.^15^ It is thought life emerged during the Hadean Eon (4.5–4.0 BYA), making the oldest accepted evidence of life dating at least half a billion years after its origin.^14^

While traces of early life are found on Earth, its origin may have occurred elsewhere and been brought to Earth (panspermia). Mars, our neighboring planet, is particularly interesting as the environmental conditions during its Noachian Period (4.1–3.7 BYA) were similar to the early Earth and, importantly, habitable.^16^ Around the same time, Late Heavy Bombardment (4.1–3.8 BYA) would have allowed for material exchange between the neighboring planets. However, the conditions on Mars changed drastically 3.6 BYA due to the loss of its atmosphere and, therefore, surface water, making the planet’s surface uninhabitable. Nevertheless, in contrast to Earth, Mars has limited tectonics and metamorphism, and so clues as to the OoL are hoped to be preserved in the Martian geological record.^17^ To this end, the search for biosignatures is a key area in OoL studies.

Biosignatures are proxies for past life. As with life, there are multiple definitions for biosignatures, each with their own shortcomings. Gillen et al. proposed that a biosignature is *a phenomenon with a known biological cause but also with any abiotic explanations explored and ruled out*.^18^ Therefore, molecular biosignatures are the residual biochemicals left over after an organism has died and decayed, or the biochemicals produced by the organism while it was alive, but not molecules that could have been formed abiotically. To this end, to classify something as a molecular biosignature, it is also important to know what molecules were present on the early planets and what abiotic processes could have created them. For example, over 90 amino acids have been detected in meteorites, and so simply finding amino acids preserved in minerals from billions of years ago does not constitute a biosignature. At the same time, it may be tempting to see a product of the polymerization of amino acids–a peptide or a proto-protein–as a biosignature. Nevertheless, peptides can be formed on clay minerals abiotically,^19^ and have been detected on meteorites.^20^ While not a biosignature, this may suggest that early peptides could be the first proto-biomolecules.

Yet, some OoL theories disregard proteins as the first molecules to emerge (as they cannot self-replicate in modern biochemistry) and imply that RNA had to arrive first.^21^ To this end, Powner et al. showed that nucleotides can be synthesized under prebiotically relevant conditions,^22^ and various routes have been found for their polymerization to RNA, including mineral-supported.^23^ However, the synthesis of nucleotides is a multistep process requiring strict condition control, which may not be attainable outside of the laboratory, further evidenced by the sparse availability of nucleotides throughout the universe.^24^ This brings us to a classic chicken-and-egg scenario, or Eigen’s paradox,^25^ where nucleic acids can only be produced by specific enzymes (proteins), which themselves can only be made by specific nucleic acids. This paradox can be resolved by a coevolution of enzymes and nucleic acids, starting with the emergence of simple peptides, followed by a cyclic evolution of the two species together.^26^ One may also argue that this cooperative evolution is evidenced by the existence of a ribosome.^27^ On the other hand, the mechanism underpinning the acetyl-coenzyme-A pathway for carbon fixation has been postulated to originate from metal sulfide minerals at the hydrothermal vents.^28,29^

Whether to discover the emergence of proto-biomolecules or to identify biosignatures from earliest lifeforms, it is essential to understand when minerals would be supporting and enabling the development of the complexity necessary for life’s origin and when they may be hindering the reactions and preserving the molecular evidence of the event.^30,31^

### Minerals as a Genetic Material

The comparison between minerals and life has often been made, as a living organism is an ordered entity that appears to have locally decreased entropy, similar to what occurs when mineral crystals are formed. Erwin Schrödinger described living organisms as “aperiodical crystals”,^32^ and Gustaf Arrhenius claimed that minerals are self-assembling, efficient reproducers capable of evolution, thus meeting the minimal definition of life.^33^

Mineral evolution has been the subject of work by Hazen and co-workers, stating that with the passing of time and the occurrence of physical, chemical, or biological processes, the diversity of minerals is expanding, and so is their ability to store structural information.^34^ While the rise of the discovery of minerals with a higher degree of information storage is evident,^35^ mineral characterization has occurred over a short period of time (of Earth’s history) and should be attributed to advances in analytical techniques and our knowledge, with further minerals and structures still to be identified. On the other hand, the appearance of more complex species through material recycling (which is also the case for nucleosynthesis) will increase overall entropy, even if decreasing it locally through crystallization or assembly.^36^ Tracing the appearance of ordered structures and the evolution of their complexity beyond a given threshold has been suggested by Marshall et al. as a sign of life itself and, therefore, a biosignature.^37^ Remarkably, some naturally occurring minerals do appear to overcome the set biotic threshold.^38^ To our best knowledge, those identified minerals are formed in the current terrestrial biosphere, but not biotically. Whether this is purely a limitation of our mineral knowledge to the samples accessible (i.e., on Earth) or whether those minerals may be indicators of habitability is yet to be explored.

**Figure 1:**
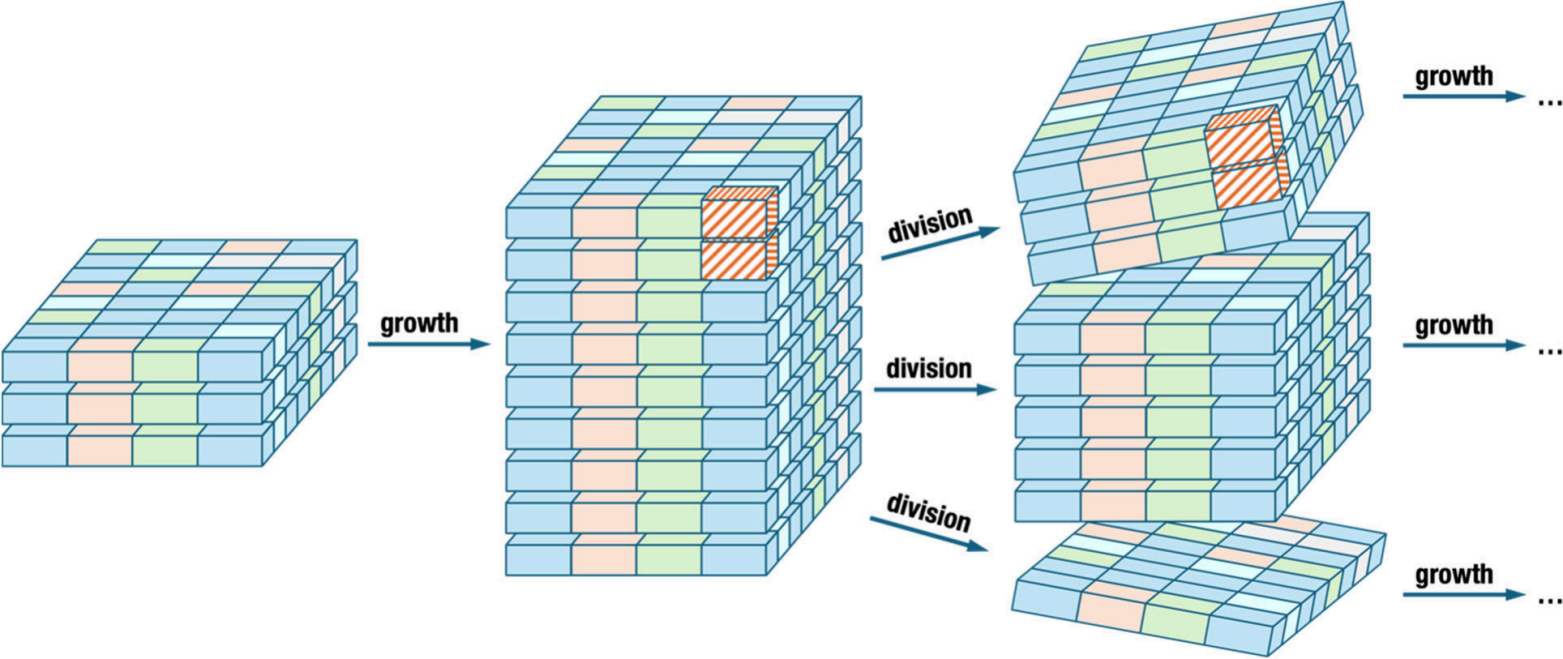
Illustration of the mineral replication process envisioned by Cairns-Smith. Clay layers are made of unit cells. Cells of varying colors represent substitutions; the dashed cell represents a new mutation emerging during growth.

The idea that minerals could have been the first living form can be attributed to Cairns-Smith, who theorized that minerals would have been the “scaffolding” that supported the formation of the first living species and was later removed as more efficient mechanisms came into place.^39^ The mineral, thanks to its ability to hold and propagate information by division and growth, would act as the first genetic material.^39^ Importantly, the system must be open, allowing for continuous crystallization. In nature, clay mineral formation is the most common continuous process, resulting in layered crystals under 2 μm in diameter. Clay minerals are susceptible to structural irregularities due to substitution and layer orientations, while the ability to template during growth and separation of layers (Figure 1) makes them good candidates for a genetic material.^39^ At first, a study by Weiss claimed experimental evidence of silicate clay replication,^40^ supporting the proposed theory; however, the study itself was nonreproducible. Similarly, the investigation of other minerals also did not show sustainable transfer of information from the original crystal.^41^

A key aspect of life is its ability to reproduce information with a high degree of accuracy. The polymers of life are large and complex (even small enzymes consisting of ∼5000 atoms), leading to an astronomical number of possible arrangements and, thus, potential errors. Therefore, these organic biomolecules require a large support system of other molecules for the genetic information to be accurately replicated. In Cairns-Smith’s theory, minerals are “primitive machines” that are necessarily made from immediately available materials and, therefore, relatively basic. As the minerals “evolve”, they can create more complex components through chemical reactions, which are then available for use in the creation of a more efficient genetic material, eventually evolving to be RNA and DNA, used by life-as-we-know-it.

### Minerals as Stepping Stones for Life

Goldschmidt and Bernal were the first to propose, separately, that minerals could have played a key role in the origin of life.^12,13^ The role of minerals is typically seen as selectively concentrating organic species, catalyzing reactions, and protecting formed molecules from the harsh environment. At the same time, organic molecules can also facilitate the precipitation of minerals, assisting in the nucleation and stabilization of crystals, favoring certain structures.^42,43^ Such organic–inorganic interactions could be seen as a prototype of a symbiotic relationship, assisting in the coevolution of mineral-organic systems.

While many minerals have been investigated in the OoL context,^44^ two groups of minerals have been a major focus for researchers: (i) metal sulfide clusters, due to their ability to fixate carbon, acting as primordial metabolism;^45^ and (ii) layered minerals, thanks to their ability to adsorb various organic species in their interlayers, protecting and concentrating them.^46^ The scope of our Account is mostly on the role of layered minerals in the formation of proto-biomolecules. Yet, we also appreciate the importance of discussion of the role of mineral clusters and ions in the emergence of metabolism, and so we urge our readers to the reviews by Muchowska et al.^47^ and Prakash et al.^48^

**Figure 2:**
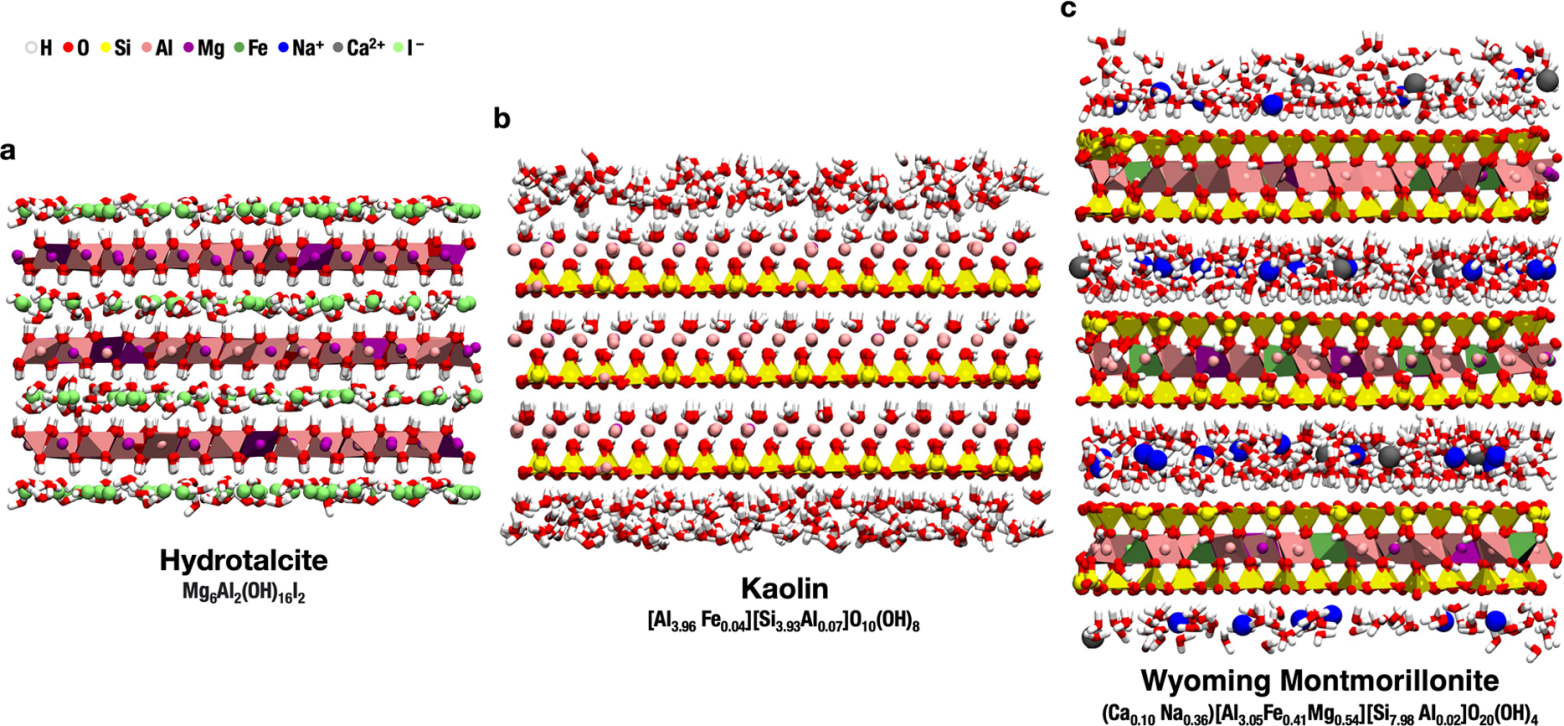
Renderings of layered mineral structures: (a) hydrotalcite, (b) kaolin, and (c) montmorillonite (a common representative of the smectite group).

Within layered minerals, smectite, kaolin-serpentinite, and hydrotalcite (layered double hydroxides) groups have attracted the most interest.^49^ These have significantly different structures (Figure 2), which give rise to very specific properties, driving their function and defining their potential role in the OoL.

Both smectites and kaolin-serpentinites are silicate clay minerals, with layers composed from tetrahedral (T) siloxane sheets and octahedral (O) hydroxide sheets. Smectites (Figure 2a) are made of TOT layers, which can feature isomorphic substitutions (e.g., octahedral Al^3+^ for Mg^2+^ or tetrahedral Si^4+^ for Fe^3+^) that create a permanent negative charge. This charge is then counterbalanced by hydrated cations in the expandable interlayer. The interlayer can be intercalated by organic species via numerous mechanisms,^50^ making these clays extremely interesting as organic material hosts. Furthermore, clay minerals can exhibit noncentrosymmetric structures, leading to the enantiomeric arrangement of the interlayer space. Kaolin-serpentines (Figure 2b) are composed of TO layers that rarely have substitutions and, therefore, no permanent charge. Instead, they have two very different exposed surfaces–hydrophobic siloxane and hydrophilic hydroxide. The hydroxide surface responds to the environmental acidity, readily deprotonating and creating a surface charge. Kaolin minerals will feature both negative and positive surfaces over a range of pHs, which allows them to interact with a wide range of organic species.^4^ In general, clay minerals are very stable species and will buffer both acidity and basicity, creating a local pH environment.^51^ They are formed by the reaction of silicate minerals and water, and so, once liquid water became available on the rocky planets, clay minerals likely became abundant.^52^ Mineralogical data on Martian soils has shown that clays are prevalent and would have formed during a habitable (i.e., water-rich) period in Martian history.^52^ With a track record of preservation of fossils of Earth, clay soils are an attractive location for the search for biosignatures by Mars missions.^53^

Layered double hydroxides (LDH, Figure 2c) do not contain silica and are comprised of octahedral sheets only. Unlike smectites, substitutions (e.g., Mg^2+^ for Al^3+^) create a positive charge, balanced by negative interlayer counterions. LDHs form in alkaline environments, such as during serpentinization reactions in alkaline hydrothermal vents.^54^ LDHs are sensitive in their metal composition and ordering to the local pH and ion gradients, and will dissolve if pH is lowered, releasing metal cations.^55^ Natural LDHs are sparse under the current oxidative environment, yet they are thought to have been common in early Earth sediments.^56^ One of the major LDH minerals of interest in this context is green rust, or fougerite,^57^ made from predominantly Fe^3+^ and Fe^2+^, which gives it electron transfer abilities and additional reactivity.^55^

#### Compartmentalization

**Figure 3:**
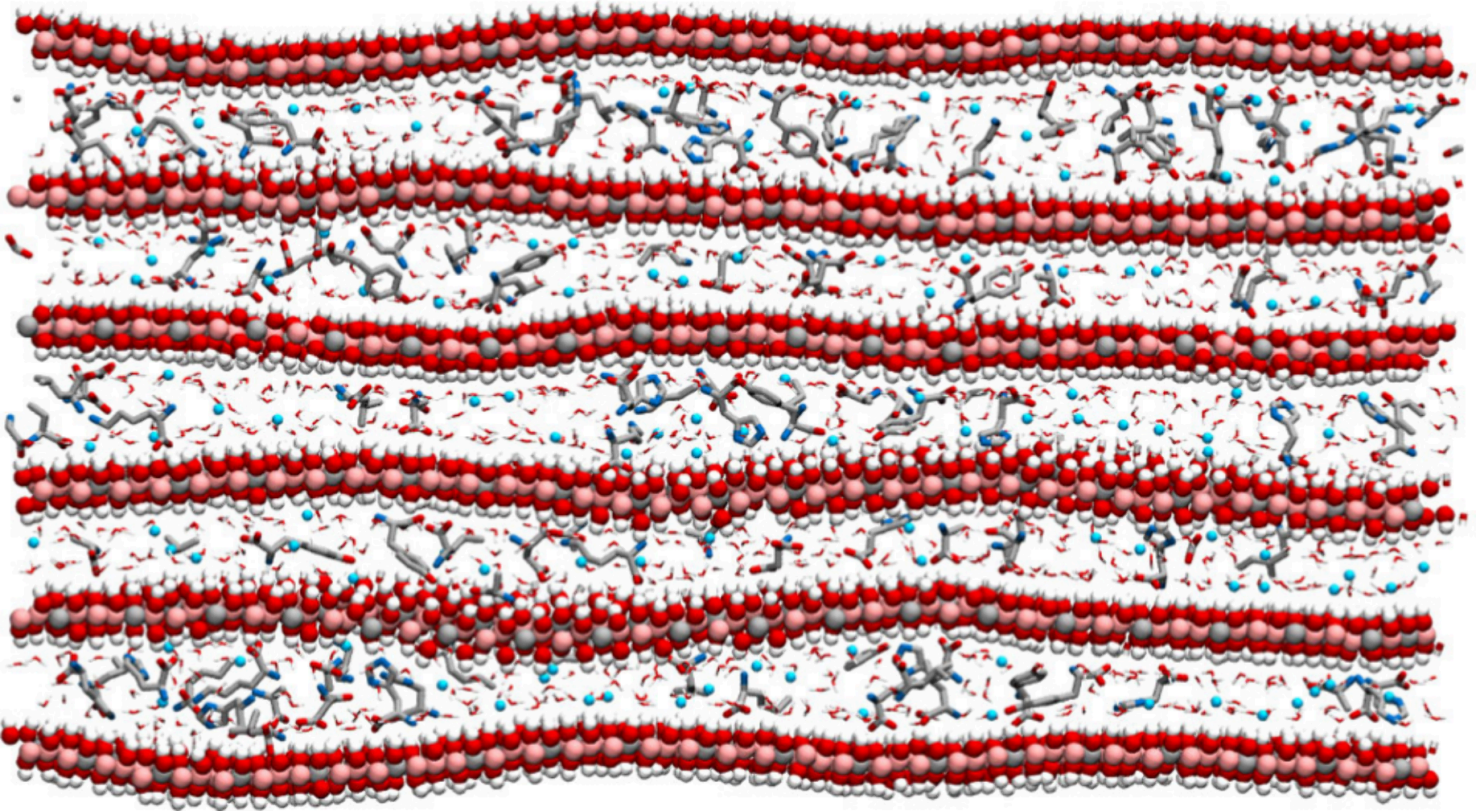
A rendering showing amino acids intercalated between LDH layers. Notably, the flexibility of the layers allows for the formation of organic-rich pockets. The simulation was carried out as part of the Erastova et al. study (previously unpublished image).^1^

Organic species, adsorbed and intercalated into the interlayer space, will be concentrated and encapsulated by the mineral layers, creating a *compartment*. Swelling silicates have been proposed as the first cells,^58^ and have been shown to form semipermeable spherules at the interface with water, mimicking cell membranes.^59^ Meanwhile, through a molecular modeling study, we were able to observe the formation of organic-rich pockets within flexible LDH layers, further condensing and coordinating amino acids (Figure 3).^1^ This softness of the layer structure is not an artifact of the modeling, but an interesting and important feature of LDHs, also documented experimentally.^57,60^ Compartmentalization is an essential step in the emergence of life, as it ensures the components are concentrated, enabling further reactions. These compartments can be seen as low entropy pockets, which are argued to be the driving component of universal evolution.^61^

#### Catalysis

Organic species adsorbed, concentrated, and encapsulated by the minerals are subjected to a new local chemical environment. One of the most widely investigated reactions, enabled by mineral surfaces, is amino acid condensation to produce peptides. Peptide formation catalyzed by silicate clays has been known for over half a century,^62^ demonstrating enantioselectivity and reliably producing long-chain polypeptides.

**Figure 4:**
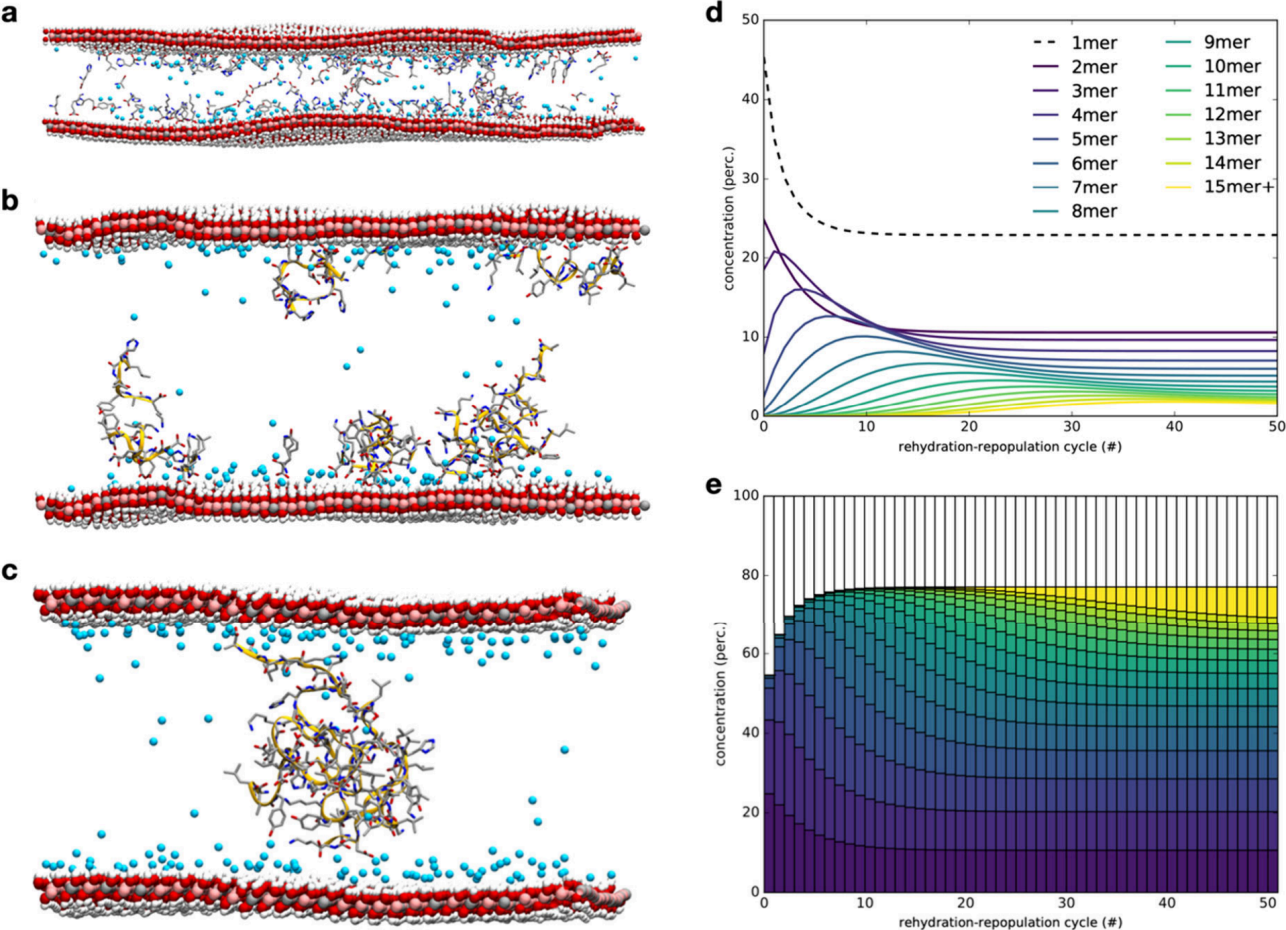
Left column shows the steps during LDH-mediated peptide formation: (a) adsorption of amino acids on the surface via C terminus; (b) polymerization and mobilization of peptides, driven by peptide agglomeration, that creates a high cumulative charge density at the C-terminus sites in contact with a less charge-dense surface; (c) hydrophobic collapse drives the formation of a folded/globular peptide, which is only lightly interacting with the surface via its C terminus. The right column shows (d) the predicted length of the peptide formed over 50 rehydration–repopulation cycles; (e) the kinetic model is based on the data derived from molecular dynamics simulations. Panels (a–c) and (e) are reproduced with permission from ref (1). Copyright 2017 Springer Nature. Panel (d) is a previously unpublished visualization of the data.

While it is important for a peptide to have a certain length to be able to fulfill a function in a biological system, the problem in the scenario of silicate-mediated peptide formation is that, upon polymerization, the formed peptide remains adsorbed on the mineral surface. This leads to the blocking of the catalytic site and makes the peptide unable to move to a new location, where it can become a part of a functioning proto-organism. In short, the formed peptide is *overprotected*. To this end, we investigated the potential for amino acid condensation reactions on hydroxide surfaces.^1,2^ The molecular modeling study demonstrated that peptide formation mediated by the LHDs is possible, and importantly, highly mobile peptides are produced (Figure 4 (a-c)). The mechanism features a stepwise elongation of the chain, notably resembling ribosome-catalyzed peptide bond formation.

Our theoretical study predicts the formation of short peptides with low yield, which was later confirmed by a laboratory study.^63^ In order to achieve an elongation of the peptide chain, we suggested employing wetting-drying cycles to power the reaction entropically and, even more crucially, to allow for the repopulation of used-up reactants and the removal of the products (Figure 4 (d, e)). This proposed mechanism highlights the importance of the open system, predicting the formation of detectable amounts of biologically relevant peptide lengths after 30+ cycles. The low yield of this reaction must also be discussed. In our modern-day efficiency-driven chemistry, it is seen as a hindrance, yet at the point of the OoL, processes occurred on the geological rather than biological time scale. And so, what is now perceived to us as unacceptably slow and low-yield is perfectly functional within the available time scales of OoL, which allow for the steady accumulation of material.

#### Selectivity

One of the essential properties of a living system is its ability to transfer information by replicating predefined sequences in a forming polymer. To this end, for a mineral to take the role in an information transfer, it must have the ability to selectively adsorb species from the environment before catalyzing polymerization. The most obvious example of selectivity is biological homochirality. Here, chiral minerals, including silicate clays, have drawn attention as a point of breaking the symmetry.^64^ While it is reasonable to assume that mineral surfaces may have led to the original enantioselectivity, it should also not be dismissed that homochirality could have emerged later at a point of selectivity for the emerged functionality.

The other consideration is the selectivity in sequestering monomers from the solution for subsequent polymerization in a way that would produce a required polymer sequence. Here, generally, are two routes: (i) the mineral acts as a template, adsorbing multiple kinds of monomers and arranging them on its surface (e.g., above substitutions or defects) in a sequence of a desired polymer, which is then generated in the following single condensation step; and (ii) the mineral acts as a transcriber of another source of information (e.g., changing environment), sequentially carrying out steps of selective adsorption and polymerization, followed by another round of adsorption and polymerization, and so on. The minerals capable of these functions are of a very different nature. For the templating, the mineral should be able to encode information in its own structure and preserve it over a prolonged period–this is a mineral type envisioned by Cairns-Smith. And indeed, multiple studies have indicated that clays are both capable of selective adsorption and polymerization, both with a strong correlation to the clays’ structure.^19,65^ On the other hand, the mineral that is capable of transcribing information should be adaptable to a changing environment, and this mineral does not need a high degree of intrinsic information storage. We could imagine such a mineral to be an LDH in a hydrothermal vent, responding in its structural changes to the available metal cations and pH gradients, able to both grow and dissolve.^54^ For such a mineral to fulfill its function, the polymerization on its surface should not be too rapid, so it allows for the changes in the mineral itself before the uptake of the monomers for the following polymerization step.

#### Protection

For both the emergence of proto-biomolecules and the preservation of biosignatures, the protection of molecules is required, yet at different levels and time scales. Organic molecules intercalated into a mineral matrix will experience a new chemical environment and be shielded from the outside one. The mineral surfaces may catalyze certain reactions but may also stabilize the species and protect them from degradation. Biosignature preservation requires very long-term protection (on a scale of billions of years) in a chemically inactive environment. For the emergence of proto-biomolecules, protection of forming species from UV radiation and hydrolysis is required on a short-term basis to allow for the polymerization reaction to occur. However, in this context, the environment must remain chemically active, as too much protection may hinder necessary reactions.

### Minerals as Vessels for Traces of Life

Clay minerals have a track record of long-term preservation of organics, making clay-rich soils of interest to Martian missions. Nevertheless, clay’s ability to readily polymerize amino acids into peptides (that are then preserved in the interlayers) creates a risk of confusing these peptides for a biosignature. In this case, one must find routes to distinguish pseudobiosignatures from true ones, that would have emerged and functioned elsewhere and only later traveled onto clay to be preserved.

Recently, peptides have been detected on silicate meteorites,^20^ demonstrating that our previous lack of identification of peptides on extraterrestrial samples may not be due to their absence but rather a technical limitation. Typically, to detect organic species on a mineral, the sample is first subjected to harsh chemical treatment to detach the organics. This treatment not only separates organics but also fragments them, providing information only on the small molecular components, here–amino acids. Currently, the only route to confidently detect a peptide on a mineral is via the enzymatic breakdown of peptide bonds.^20^ While exciting and enabling, this approach is not attainable on extraterrestrial missions due to the risk of contamination by biological species, and therefore, any samples would have to first be returned to Earth.

**Figure 5:**
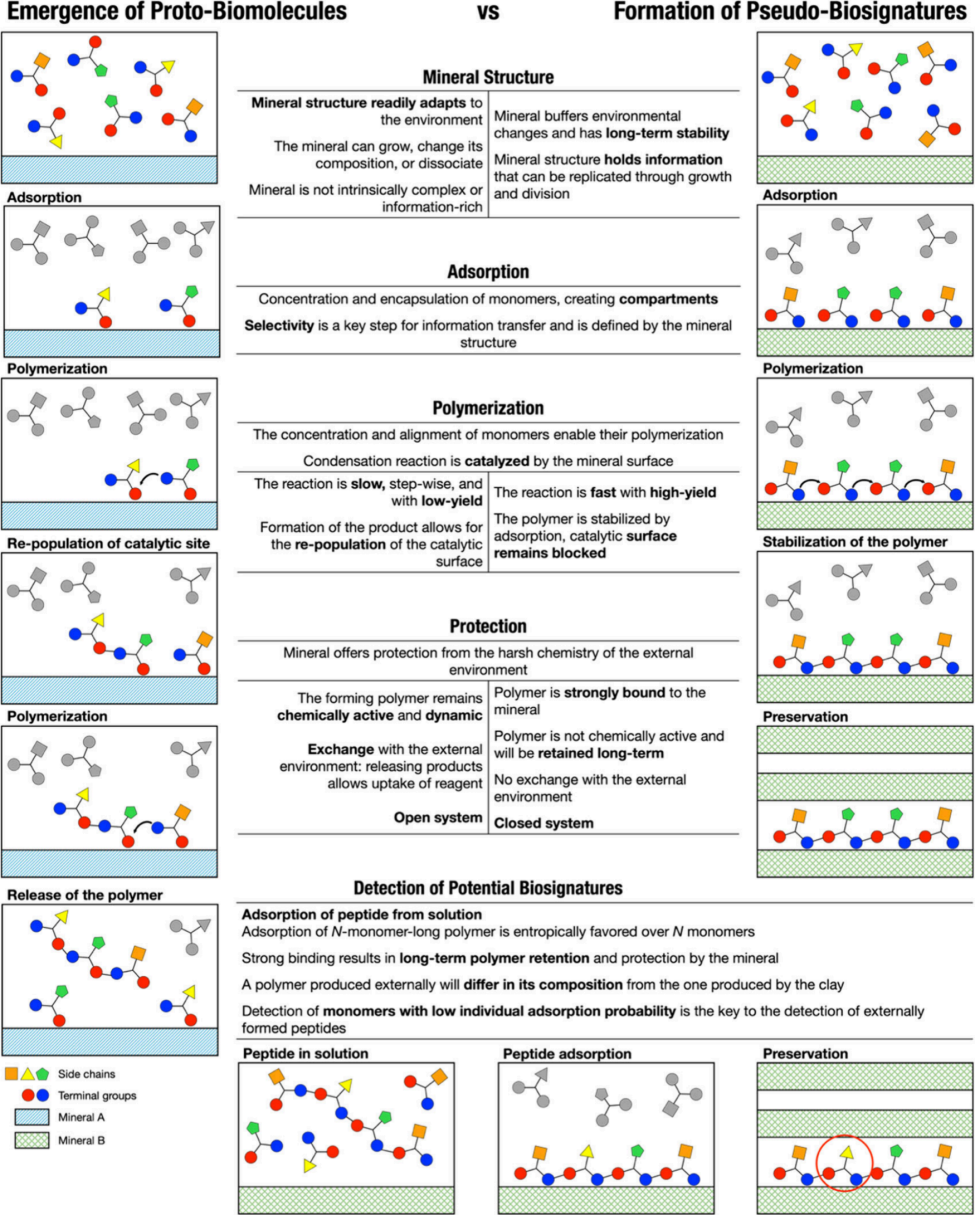
Graphical summary of (left column) the formation of a potential proto-biomolecule, catalyzed by a mineral capable of transcribing information from the environment to the forming polymer, producing a peptide that is released into the environment; (right column) the formation of a pseudobiosignature, where monomer adsorption selectivity is driven by the mineral structure, producing a polymer strongly bound to the surface, that will remain protected for long-term preservation; and (bottom) a route to the identification of pseudobiosignatures and a potential proto-biomolecule preserved by a mineral. The process depicted in the left column is a reinterpretation of the mechanism, originally suggested by Erastova et al.^1^

In view of future sample return missions, one must find a route to discard the samples containing pseudobiosignatures produced by a mineral. Ideally, we must do so by only knowing the retained amino acid distribution and the mineral structure, as this is the data we realistically could obtain.^66^ To this end, the knowledge of the selectivity of a given mineral toward adsorbed species would hold the key, ultimately suggesting the composition of a polymer generated by this mineral surface. Anything matching this composition can be confidently discarded as a pseudobiosignature. On the other hand, the presence of amino acids that are unlikely to be adsorbed by this mineral under the given conditions will highlight that those had to arrive bound to some other species with favorable adsorption, such as in the form of a peptide. While it is not possible to then confidently conclude if this peptide is a proto-biomolecule, it would suggest that there is (or was) another environment in proximity, where this molecule was produced and then released. In Figure 5, we summarize the processes and mineral types involved in the emergence of a potential proto-biomolecule and the formation of pseudobiosignature; we also suggest an approach to the identification of a potential biosignature preserved on a mineral.

### Molecular Modeling to Bridge Across Time and Space Scales

The OoL research unavoidably faces two major obstacles–the time and space scales. The emergence of life did not happen overnight, with the processes that converted geochemistry to biochemistry having geological time scales available to their service. Yet, neither our lifetime nor research laboratory constraints permit us to attempt such slow low-yield experiments. Shortcuts must be taken, which include carrying out studies with elevated sampling or accelerated dynamics (e.g., increasing species concentration and system temperature), thus putting the study outside of realistic OoL conditions. On the other hand, the location of OoL is not constrained, and even with current sensational developments in space travel, we are unable to travel to the locations or obtain samples in necessary quantities. To this end, we set up experiments on Earth that are proxies to the environments and mineralogy of other planets.

The rapidly developing area of molecular modeling gives us another tool to close these gaps. Molecular modeling is a set of theoretical techniques that utilize the laws of physics to allow us to test hypothetical scenarios, even those unattainable in the lab.^67^ Molecular dynamics simulations, as used routinely by our group, provide atomistic-level information on the mineral-organic interactions, allowing us to examine processes at the mineral interface and compare probabilities of numerous potential events. Such studies allowed us to constrain the conditions for the LDH-supported peptide formation, where 30+ rehydration-repopulation cycles are necessary to obtain a peptide with a functional length. While this could not be (and has not been) predicted by a laboratory study alone, in the context of early Earth, such a process could happen during less than the two-month period at a shallow-sea hydrothermal vent.^68^ The value of a theoretical prediction is also in its ability to minimize search space and highlight future laboratory experiments to further validate the hypothesis.

The ability of modeling methods to generate and examine hypothetical systems makes them well poised for the study of, for example, past planetary conditions or unreachable extraterrestrial minerals. It is possible to develop models of hypothetical minerals based on our knowledge of mineralogy and guided by spectroscopic readings from the planet, even if sparse. Where multiple structures can be designed from the available data, modeling allows to test and discard unlikely structures, narrowing down the search space. To this end, we have recently developed *ClayCode*,^69^ which assists in the setup and preparation of molecular dynamics simulations of layered minerals. Above all, the code ensures that the systems are truthfully representative of the finest structural details of actual physical minerals.^3^ Therefore, the code now enables us to examine the effect of the structures of Martian clays on the adsorption, selectivity, and retention of amino acids.^70^ In agreement with experimental work,^65^ we observe the selectivity of individual clays. This large-scale modeling study provides unambiguous statistics that are necessary to predict the composition of peptides, i.e., pseudobiosignatures, that would be on these clays. Furthermore, the study highlights the importance of accounting for the unique ionic compositions of Martian soils when making predictions. While the current theoretical study used well-characterized nontronites found on Earth, the reproducibility of these results in the laboratory will confirm the reliability of molecular modeling as a tool for the identification of pseudobiomolecules in extraterrestrial settings.

## Conclusion

The quest to identify the OoL mechanisms that enabled the transition from geochemistry to biochemistry has led to a wealth of scientific discoveries and brought humankind to space. But the answer to the question of the origin of life is yet to be found. While many theories agree on the importance of minerals for the emergence of life, the actual function of the mineral is debated — from being the first genetic material, to supporting enantioselectivity, to acting as a catalyst for polymerization reactions, to protecting organic molecules from harsh environments (to name a few).

With this Account, we critically review the current state of knowledge on the role of mineral surfaces and identify the key descriptors for such minerals to support the emergence and preservation of proto-biomolecules. Furthermore, we suggest a route to identify a biosignature-lookalike that could be naturally formed on a mineral surface. Finally, recognizing that the time scales available for the processes leading to life’s origin and that obtaining samples from extraterrestrial locations are (currently) both unattainable, we discuss the contribution that molecular modeling can make in investigating those scenarios. We hope that this Account will be a stepping-stone to support the discovery of the mechanisms of the formation of the first proto-biomolecules and, consequently, their detection as biosignatures.

## References

[ref1] Erastova V.; Degiacomi M. T.; Fraser D. G.; Greenwell H. C. Mineral Surface Chemistry Control for Origin of Prebiotic Peptides. Nat. Commun. 2017, 8 (1), 1–9. 10.1038/s41467-017-02248-y. 29229963 PMC5725419

[ref2] Grégoire B.; Erastova V.; Geatches D. L.; Clark S. J.; Greenwell H. C.; Fraser D. G. Insights into the Behaviour of Biomolecules on the Early Earth: The Concentration of Aspartate by Layered Double Hydroxide Minerals. Geochim. Cosmochim. Acta 2016, 176, 239–258. 10.1016/j.gca.2015.12.026.

[ref3] Nuruzade O.; Abdullayev E.; Erastova V. Organic-Mineral Interactions under Natural Conditions: A Computational Study of Flavone Adsorption on Smectite Clay. J. Phys. Chem. C 2023, 127 (27), 13167–13177. 10.1021/acs.jpcc.3c00174.

[ref4] Zhao R.; Xue H.; Lu S.; Greenwell H. C.; Erastova V. Revealing Crucial Effects of Reservoir Environment and Hydrocarbon Fractions on Fluid Behaviour in Kaolinite Pores. Chemical Engineering Journal 2024, 489, 151362 10.1016/j.cej.2024.151362.

[ref5] Raulin-Cerceau F. Historical Review of the Origin of Life and Astrobiology. Origins 2005, 6, 15–33. 10.1007/1-4020-2522-X_3.

[ref6] Walker S. I.; Packard N.; Cody G. D. Re-Conceptualizing the Origins of Life. Philosophical Transactions of the Royal Society A: Mathematical, Physical and Engineering Sciences 2017, 375 (2109), 20160337 10.1098/rsta.2016.0337. PMC5686397 29133439

[ref7] Gánti T. Biogenesis Itself. J. Theor. Biol. 1997, 187 (4), 583–593. 10.1006/jtbi.1996.0391. 9299301

[ref8] Preiner M.; Asche S.; Becker S.; Betts H. C.; Boniface A.; Camprubi E.; Chandru K.; Erastova V.; Garg S. G.; Khawaja N.; Kostyrka G.; Machné R.; Moggioli G.; Muchowska K. B.; Neukirchen S.; Peter B.; Pichlhöfer E.; Radványi Á.; Rossetto D.; Salditt A.; Schmelling N. M.; Sousa F. L.; Tria F. D. K.; Vörös D.; Xavier J. C. The Future of Origin of Life Research: Bridging Decades-Old Divisions. Life 2020, 10 (3), 20 10.3390/life10030020. 32110893 PMC7151616

[ref9] Miller S. L. A Production of Amino Acids under Possible Primitive Earth Conditions. Science (1979) 1953, 117 (3046), 528–529. 10.1126/science.117.3046.528. 13056598

[ref10] Pizzarello S.; Cooper G. W.; Flynn G. J. The Nature and Distribution of the Organic Material in Carbonaceous Chondrites and Interplanetary Dust Particles. In Meteorites and the Early Solar System II; Lauretta D. S., McSween H. Y. Jr., Eds.; University of Arizona Press: Tucson, 2006; pp 625–651.

[ref11] Cornish-Bowden A.; Cárdenas M. L. Contrasting Theories of Life: Historical Context, Current Theories. In Search of an Ideal Theory. Biosystems 2020, 188, 104063 10.1016/j.biosystems.2019.104063. 31715221

[ref12] Bernal J. D. The Physical Basis of Life. Proceedings of the Physical Society. Section A 1949, 62 (9), 537 10.1088/0370-1298/62/9/301.

[ref13] Goldschmidt V. M. Geochemical Aspects of the Origin of Complex Organic Molecules on the Earth, as Precursors to Organic Life. In New Biology; Pirie N. W., Johnson M. L., Abercrombie M., Eds.; Penguin Books, 1952; Vol. 12, pp 97–105.

[ref14] Cavalazzi B.; Hickman-Lewis K.; Brack A.; Cady S. L. Earliest Traces of Life as a Window on Life’s Origins. Advances in Astrobiology and Biogeophysics 2021, 227–254. 10.1007/978-3-030-81039-9_10.

[ref15] McMahon S.; Jordan S. F. A Fundamental Limit to the Search for the Oldest Fossils. Nat. Ecol Evol 2022, 6 (7), 832–834. 10.1038/s41559-022-01777-0. 35577985

[ref16] Ehlmann B. L.; Anderson F. S.; Andrews-Hanna J.; Catling D. C.; Christensen P. R.; Cohen B. A.; Dressing C. D.; Edwards C. S.; Elkins-Tanton L. T.; Farley K. A.; Fassett C. I.; Fischer W. W.; Fraeman A. A.; Golombek M. P.; Hamilton V. E.; Hayes A. G.; Herd C. D. K.; Horgan B.; Hu R.; Jakosky B. M.; Johnson J. R.; Kasting J. F.; Kerber L.; Kinch K. M.; Kite E. S.; Knutson H. A.; Lunine J. I.; Mahaffy P. R.; Mangold N.; McCubbin F. M.; Mustard J. F.; Niles P. B.; Quantin-Nataf C.; Rice M. S.; Stack K. M.; Stevenson D. J.; Stewart S. T.; Toplis M. J.; Usui T.; Weiss B. P.; Werner S. C.; Wordsworth R. D.; Wray J. J.; Yingst R. A.; Yung Y. L.; Zahnle K. J. The Sustainability of Habitability on Terrestrial Planets: Insights, Questions, and Needed Measurements from Mars for Understanding the Evolution of Earth-like Worlds. J. Geophys Res. Planets 2016, 121 (10), 1927–1961. 10.1002/2016JE005134.

[ref17] Vago J. L.; Westall F.; Coates A. J.; Jaumann R.; Korablev O.; Ciarletti V.; Mitrofanov I.; Josset J. L.; De Sanctis M. C.; Bibring J. P.; Rull F.; Goesmann F.; Steininger H.; Goetz W.; Brinckerhoff W.; Szopa C.; Raulin F.; Edwards H. G. M.; Whyte L. G.; Fairén A. G.; Bridges J.; Hauber E.; Ori G. G.; Werner S.; Loizeau D.; Kuzmin R. O.; Williams R. M. E.; Flahaut J.; Forget F.; Rodionov D.; Svedhem H.; Sefton-Nash E.; Kminek G.; Lorenzoni L.; Joudrier L.; Mikhailov V.; Zashchirinskiy A.; Alexashkin S.; Calantropio F.; Merlo A.; Poulakis P.; Witasse O.; Bayle O.; Bayón S.; Meierhenrich U.; Carter J.; García-Ruiz J. M.; Baglioni P.; Haldemann A.; Ball A. J.; Debus A.; Lindner R.; Haessig F.; Monteiro D.; Trautner R.; Voland C.; Rebeyre P.; Goulty D.; Didot F.; Durrant S.; Zekri E.; Koschny D.; Toni A.; Visentin G.; Zwick M.; Van Winnendael M.; Azkarate M.; Carreau C.; Westall F.; Bibring J.-P.; Vago J. L.; Korablev O. Habitability on Early Mars and the Search for Biosignatures with the ExoMars Rover. Astrobiology 2017, 17 (6–7), 471–510. 10.1089/ast.2016.1533. 31067287 PMC5685153

[ref18] Gillen C.; Jeancolas C.; McMahon S.; Vickers P. The Call for a New Definition of Biosignature. Astrobiology 2023, 23 (11), 1228–1237. 10.1089/ast.2023.0010. 37819715

[ref19] Asche S.; Pow R. W.; Mehr H. M.; Cooper G. J. T.; Sharma A.; Cronin L. Evidence of Selection in Mineral Mediated Polymerization Reactions Executed in a Robotic Chemputer System. ChemSystemsChem. 2024, 6, e202400006 10.1002/syst.202400006.

[ref20] Lange J.; Djago F.; Eddhif B.; Remaury Q. B.; Ruf A.; Leitner N. K. V.; Hendecourt L. L. S.; Danger G.; Rodier C. G.; Papot S.; Poinot P. A Novel Proteomics-Based Strategy for the Investigation of Peptide Sequences in Extraterrestrial Samples. J. Proteome Res. 2021, 20 (2), 1444 10.1021/acs.jproteome.0c00700. 33078610

[ref21] Gilbert W. Origin of Life: The RNA World. Nature 1986, 319 (6055), 618–618. 10.1038/319618a0.

[ref22] Powner M. W.; Gerland B.; Sutherland J. D. Synthesis of Activated Pyrimidine Ribonucleotides in Prebiotically Plausible Conditions. Nature 2009, 459 (7244), 239–242. 10.1038/nature08013. 19444213

[ref23] Ferris J. P.; Hill A. R.; Liu R.; Orgel L. E. Synthesis of Long Prebiotic Oligomers on Mineral Surfaces. Nature 1996, 381 (6577), 59–61. 10.1038/381059a0. 8609988

[ref24] Bregestovski P. D. RNA World”, a Highly Improbable Scenario of the Origin and Early Evolution of Life on Earth. J. Evol Biochem Physiol 2015, 51 (1), 72–84. 10.1134/S0022093015010111.

[ref25] Eigen M. Selforganization of Matter and the Evolution of Biological Macromolecules. Naturwissenschaften 1971, 58 (10), 465–523. 10.1007/BF00623322. 4942363

[ref26] Eigen M.; Schuster P. Stages of Emerging Life -Five Principles of Early Organization. J. Mol. Evol 1982, 19 (1), 47–61. 10.1007/BF02100223. 7161810

[ref27] Fried S. D.; Fujishima K.; Makarov M.; Cherepashuk I.; Hlouchova K. Peptides before and during the Nucleotide World: An Origins Story Emphasizing Cooperation between Proteins and Nucleic Acids. J. R. Soc. Interface 2022, 19 ( (187), ), 10.1098/rsif.2021.0641. PMC8833103 35135297

[ref28] Russell M. J.; Martin W. The Rocky Roots of the Acetyl-CoA Pathway. Trends Biochem. Sci. 2004, 29 (7), 358–363. 10.1016/j.tibs.2004.05.007. 15236743

[ref29] Preiner M.; Igarashi K.; Muchowska K. B.; Yu M.; Varma S. J.; Kleinermanns K.; Nobu M. K.; Kamagata Y.; Tüysüz H.; Moran J.; Martin W. F. A Hydrogen-Dependent Geochemical Analogue of Primordial Carbon and Energy Metabolism. Nature Ecology & Evolution 2020 4:4 2020, 4 (4), 534–542. 10.1038/s41559-020-1125-6. 32123322

[ref30] Saha R.; Kao W. L.; Malady B.; Heng X.; Chen I. A. Effect of Montmorillonite K10 Clay on RNA Structure and Function. Biophys. J. 2024, 123 (4), 451–463. 10.1016/j.bpj.2023.11.002. 37924206 PMC10912936

[ref31] dos Santos R.; Patel M.; Cuadros J.; Martins Z. Influence of Mineralogy on the Preservation of Amino Acids under Simulated Mars Conditions. Icarus 2016, 277, 342–353. 10.1016/j.icarus.2016.05.029.

[ref32] Schrödinger E.; Penrose R. Mutations. What is Life?; Cambridge University Press, 1992; pp 32–45. 10.1017/CBO9781139644129.006.

[ref33] Arrhenius G. O. Crystals and Life. Helv. Chim. Acta 2003, 86 (5), 1569–1586. 10.1002/hlca.200390135.

[ref34] Hazen R. M.; Papineau D.; Bleeker W.; Downs R. T.; Ferry J. M.; McCoy T. J.; Sverjensky D. A.; Yang H. Mineral Evolution. Am. Mineral. 2008, 93 (11–12), 1693–1720. 10.2138/am.2008.2955.

[ref35] Krivovichev S. V.; Krivovichev V. G.; Hazen R. M.; Aksenov S. M.; Avdontceva M. S.; Banaru A. M.; Gorelova L. A.; Ismagilova R. M.; Kornyakov I. V.; Kuporev I. V.; Morrison S. M.; Panikorovskii T. L.; Starova G. L. Structural and Chemical Complexity of Minerals: An Update. Mineral Mag 2022, 86 (2), 183–204. 10.1180/mgm.2022.23.

[ref36] Frautschi S. Entropy in an Expanding Universe. Science (1979) 1982, 217 (4560), 593–599. 10.1126/science.217.4560.593. 17817517

[ref37] Marshall S. M.; Mathis C.; Carrick E.; Keenan G.; Cooper G. J. T.; Graham H.; Craven M.; Gromski P. S.; Moore D. G.; Walker S. I.; Cronin L. Identifying Molecules as Biosignatures with Assembly Theory and Mass Spectrometry. Nat. Commun. 2021, 12 (1), 1–9. 10.1038/s41467-021-23258-x. 34031398 PMC8144626

[ref38] Hazen R. M.; Burns P. C.; Cleaves H. J.; Downs R. T.; Krivovichev S. V.; Wong M. L. Molecular Assembly Indices of Mineral Heteropolyanions: Some Abiotic Molecules Are as Complex as Large Biomolecules. J. R Soc. Interface 2024, 21 (211), 20230632 10.1098/rsif.2023.0632. 38378136 PMC10878807

[ref39] Cairns-Smith A. G. Genetic Takeover and the Mineral Origins of Life; Cambridge University Press, 1982.

[ref40] Weiss A. Replication and Evolution in Inorganic Systems. Angewandte Chemie International Edition in English 1981, 20 (10), 850–860. 10.1002/anie.198108501.

[ref41] Bullard T.; Freudenthal J.; Avagyan S.; Kahr B. Test of Cairns-Smith’s ‘Crystals-as-Genes’ Hypothesis. Faraday Discuss. 2007, 136 (0), 231–245. 10.1039/b616612c. 17955812

[ref42] Bontognali T. R. R.; Martinez-Ruiz F.; McKenzie J. A.; Bahniuk A.; Anjos S.; Vasconcelos C. Smectite Synthesis at Low Temperature and Neutral PH in the Presence of Succinic Acid. Appl. Clay Sci. 2014, 101, 553–557. 10.1016/j.clay.2014.09.018.

[ref43] Xu S.; Zhao J.; Yu Q.; Qiu X.; Sasaki K. Effect of Natural Organic Matter Model Compounds on the Structure Memory Effect of Different Layered Double Hydroxides. ACS Earth Space Chem. 2019, 3 (10), 2175–2189. 10.1021/acsearthspacechem.9b00175.

[ref44] Hazen R. M. Paleomineralogy of the Hadean Eon: A Preliminary Species List. Am. J. Sci. 2013, 313 (9), 807–843. 10.2475/09.2013.01.

[ref45] Preiner M.; Xavier J. C.; do Nascimento Vieira A.; Kleinermanns K.; Allen J. F.; Martin W. F. Catalysts, Autocatalysis and the Origin of Metabolism. Interface Focus 2019, 9 (6), 20190072 10.1098/rsfs.2019.0072. 31641438 PMC6802133

[ref46] Duval S.; Branscomb E.; Trolard F.; Bourrié G.; Grauby O.; Heresanu V.; Schoepp-Cothenet B.; Zuchan K.; Russell M. J.; Nitschke W. On the Why’s and How’s of Clay Minerals’ Importance in Life’s Emergence. Appl. Clay Sci. 2020, 195, 105737 10.1016/j.clay.2020.105737.

[ref47] Muchowska K. B.; Varma S. J.; Moran J. Nonenzymatic Metabolic Reactions and Life’s Origins. Chem. Rev. 2020, 120 (15), 7708–7744. 10.1021/acs.chemrev.0c00191. 32687326

[ref48] Prakash M.; Weber J. M.; Rodriguez L. E.; Sheppard R. Y.; Barge L. M. Database on Mineral Mediated Carbon Reduction: Implications for Future Research. Int. J. Astrobiol 2022, 21 (6), 423–440. 10.1017/S1473550422000052.

[ref49] Cleaves H. J. II; Michalkova Scott A.; Hill F. C.; Leszczynski J.; Sahai N.; Hazen R. Mineral-Organic Interfacial Processes: Potential Roles in the Origins of Life. Chem. Soc. Rev. 2012, 41 (16), 5502–5525. 10.1039/c2cs35112a. 22743683

[ref50] Underwood T.; Erastova V.; Greenwell H. C. Wetting Effects and Molecular Adsorption at Hydrated Kaolinite Clay Mineral Surfaces. J. Phys. Chem. C 2016, 120 (21), 11433–11449. 10.1021/acs.jpcc.6b00187.

[ref51] Jeon I.; Nam K. Change in the Site Density and Surface Acidity of Clay Minerals by Acid or Alkali Spills and Its Effect on PH Buffering Capacity. Sci. Rep 2019, 9 (1), 1–10. 10.1038/s41598-019-46175-y. 31285476 PMC6614462

[ref52] Du P.; Yuan P.; Liu J.; Ye B. Clay Minerals on Mars: An up-to-Date Review with Future Perspectives. Earth Sci. Rev. 2023, 243, 104491 10.1016/j.earscirev.2023.104491.

[ref53] Ehlmann B. L.; Mustard J. F.; Fassett C. I.; Schon S. C.; Head J. W.; Des Marais D. J.; Grant J. A.; Murchie S. L. Clay Minerals in Delta Deposits and Organic Preservation Potential on Mars. Nat. Geosci 2008, 1 (6), 355–358. 10.1038/ngeo207.

[ref54] Martin W.; Baross J.; Kelley D.; Russell M. J. Hydrothermal Vents and the Origin of Life. Nature Reviews Microbiology 2008 6:11 2008, 6 (11), 805–814. 10.1038/nrmicro1991. 18820700

[ref55] Usman M.; Byrne J. M.; Chaudhary A.; Orsetti S.; Hanna K.; Ruby C.; Kappler A.; Haderlein S. B. Magnetite and Green Rust: Synthesis, Properties, and Environmental Applications of Mixed-Valent Iron Minerals. Chem. Rev. 2018, 118 (7), 3251–3304. 10.1021/acs.chemrev.7b00224. 29465223

[ref56] Barge L. M.; Price R. E. Diverse Geochemical Conditions for Prebiotic Chemistry in Shallow-Sea Alkaline Hydrothermal Vents. Nat. Geosci 2022, 15 (12), 976–981. 10.1038/s41561-022-01067-1.

[ref57] Duval S.; Baymann F.; Schoepp-Cothenet B.; Trolard F.; Bourrié G.; Grauby O.; Branscomb E.; Russell M. J.; Nitschke W. Fougerite: The Not so Simple Progenitor of the First Cells. Interface Focus 2019, 9 (6), 20190063 10.1098/rsfs.2019.0063. 31641434 PMC6802129

[ref58] Smith J. V.; Arnold F. P.; Parsons I.; Lee M. R. Biochemical Evolution III: Polymerization on Organophilic Silica-Rich Surfaces, Crystal-Chemical Modeling, Formation of First Cells, and Geological Clues. Proc. Natl. Acad. Sci. U. S. A. 1999, 96 (7), 3479–3485. 10.1073/pnas.96.7.3479. 10097060 PMC34290

[ref59] Subramaniam A. B.; Wan J.; Gopinath A.; Stone H. A. Semi-Permeable Vesicles Composed of Natural Clay. Soft Matter 2011, 7 (6), 2600–2612. 10.1039/c0sm01354d.

[ref60] Hall D. S.; Lockwood D. J.; Bock C.; MacDougall B. R. Nickel Hydroxides and Related Materials: A Review of Their Structures, Synthesis and Properties. Proceedings of the Royal Society A: Mathematical, Physical and Engineering Sciences 2015, 471 (2174), 20140792 10.1098/rspa.2014.0792. PMC4309132 25663812

[ref61] Wong M. L.; Cleland C. E.; Arend D.; Bartlett S.; Cleaves H. J.; Demarest H.; Prabhu A.; Lunine J. I.; Hazen R. M. On the Roles of Function and Selection in Evolving Systems. Proc. Natl. Acad. Sci. U. S. A. 2023, 120 (43), e2310223120 10.1073/pnas.2310223120. 37844243 PMC10614609

[ref62] Degens E. T.; Matheja J.; Jackson T. A. Template Catalysis: Asymmetric Polymerization of Amino-Acids on Clay Minerals. Nature 1970, 227 (5257), 492–493. 10.1038/227492a0. 5428467

[ref63] Grégoire B.; Greenwell H. C.; Fraser D. G. Peptide Formation on Layered Mineral Surfaces: The Key Role of Brucite-like Minerals on the Enhanced Formation of Alanine Dipeptides. ACS Earth Space Chem. 2018, 2 (8), 852–862. 10.1021/acsearthspacechem.8b00052.

[ref64] Zaia D. A. M.; Zaia C. T. B. V. A Few Experimental Suggestions Using Minerals to Obtain Peptides with a High Concentration of L-Amino Acids and Protein Amino Acids. Symmetry (Basel) 2020, 12 (12), 2046 10.3390/sym12122046.

[ref65] Millman E.; Chatterjee A.; Parker K. M.; Catalano J. G. Cation Exchange to Montmorillonite Induces Selective Adsorption of Amino Acids. Geochim. Cosmochim. Acta 2024, 372, 181 10.1016/j.gca.2024.02.020.

[ref66] Fkiri R.; Timoumi R.; Rioland G.; Poinot P.; Baron F.; Gregoire B.; Geffroy-Rodier C. Gas Chromatography Fingerprint of Martian Amino Acids before Analysis of Return Samples. Chemosensors 2023, 11 (2), 76 10.3390/chemosensors11020076.

[ref67] OoLEN; Asche S.; Bautista C.; Boulesteix D.; Champagne-Ruel A.; Mathis C.; Markovitch O.; Peng Z.; Adams A.; Dass A. V.; Buch A.; Camprubi E.; Colizzi E. S.; Colón-Santos S.; Dromiack H.; Erastova V.; Garcia A.; Grimaud G.; Halpern A.; Harrison S. A.; Jordan S. F.; Jia T. Z.; Kahana A.; Kolchinsky A.; Moron-Garcia O.; Mizuuchi R.; Nan J.; Orlova Y.; Pearce B. K. D.; Paschek K.; Preiner M.; Pinna S.; Rodríguez-Román E.; Schwander L.; Sharma S.; Smith H. B.; Vieira A.; Xavier J. C. What It Takes to Solve the Origin(s) of Life: An Integrated Review of Techniques. arXiv 2023, 10.48550/arXiv.2308.11665.

[ref68] Barge L. M.; Price R. E. Diverse Geochemical Conditions for Prebiotic Chemistry in Shallow-Sea Alkaline Hydrothermal Vents. Nat. Geosci 2022, 15 (12), 976–981. 10.1038/s41561-022-01067-1.

[ref69] Pollak H.; Degiacomi M. T.; Erastova V. Modelling realistic clay systems with ClayCode. arXiv preprint arXiv:2407.18882 (2024). 10.1021/acs.jctc.4c00987 PMC11562070 39404473

[ref70] Pollak H.; Erastova V. Understanding Interactions between Amino Acids and Nontronite Clays: What Is the Effect of Ions? Goldschmidt 2023 Conference; Goldschmidt, 2023.

